# Supplementary material for: Balance of mechanical forces drives endothelial gap formation and may facilitate cancer and immune-cell extravasation
Source: PLoS Comput Biol. 2019 May 2;15(5):e1006395. doi: 10.1371/journal.pcbi.1006395 (PMC6497229; doi:10.1371/journal.pcbi.1006395)
Supplement: S1 Table — (PDF) [file pcbi.1006395.s002.pdf]

Parameter	Symbol	Value	Source
Medium drag coefficient	ξ	$4.1 \cdot 10^{-3}$ (kg/s)	[15]
Membrane stiffness	K_{memb}	$2.5 \cdot 10^{-3}$ (kg/s^2)	[16]
Stress fiber stiffness	K_{sf}	$1.25 \cdot 10^{-4}$ (kg/s^2)	[15]
Rotational spring constant	K_{bend}	$7.5 \cdot 10^{-17}$ (Nm)	[16]
Membrane viscosity	η_{memb}	$1.109 \cdot 10^{-3}$ (kg/s)	[16]
Stress fiber viscosity	η_{sf}	$1.109 \cdot 10^{-3}$ (kg/s)	[16]
Force to normalize parameters in unbinding law	F^0	0.008 (nN)	Adjusted from [4]
Non-dimensionalized force of catch curve in unbinding law	θ_c	0.01	Adjusted from [4]
Non-dimensionalized force of slip curve in unbinding law	θ_s	4	Adjusted from [4]
Unbinding rate coefficient for catch curve	k_c^0	0.27 s^{-1}	Adjusted from [4]
Unbinding rate coefficient for slip curve	k_s^0	0.27 s^{-1}	Adjusted from [4]
Binding rate for adhesions at maximum distance	k_{on}^0	15.3 $(\mu m^2/(mol \cdot s))$	Estimated from unbinding law
Binding rate for adhesion reinforcement at zero force	k_{reinf}^0	11.5 $(\mu m^2/(mol \cdot s))$	Estimated from unbinding law
Adhesion complex density	ρ_{adh}	21 $(mol/\mu m^2)$	[20]
Limit distance for cadherin binding	L_{bind}^{limit}	0.95 (μm)	Estimated
Force constant for reinforcement curve	λ_{reinf}	10 $nN)$	Adjusted from unbinding law
Force threshold to stop applying reinforcement	F_{reinf}^{limit}	0.06 (nN)	Adjusted from unbinding law
Adhesion complex stiffness constant per bond	K_{adh}^0	$2 \cdot 10^{-4}$ (kg/s)	Estimated
Adhesion complex equilibrium length	L_{adh}^0	0.1 (μm)	[14]
Maximum number of cadherins per clutch	n_c^{max}	8	Estimated
Maximum force due to radial contraction	F_{Radial}	0.775 (nN)	Adjusted from [13]
Maximum force due to cortical tension	F_{cortex}	0.025 (nN)	[19]
Maximum force due to protrusion	F_{Prot}	0.08 (nN)	Estimated
Force recalculation time for radial force	t_{Radial}^{Force}	25 min	Estimated
Force recalculation time for cortical force	t_{Cortex}^{Force}	25 min	Estimated
Force recalculation time for protrusive force	t_{Prot}^{Force}	25 min	Estimated
Force transition time	$t_{Transition}^{Force}$	2 min	Estimated
Number of nodes with similar radial force	n_{Radial}^{Force}	5	Estimated
Number of nodes with similar cortical force	n_{Cortex}^{Force}	10	Estimated
Number of nodes with similar protrusive force	n_{Prot}^{Force}	20	Estimated
Force activation probability for radial force	p_{Radial}^{Force}	0.01	Estimated
Force activation probability for cortical force	p_{Cortex}^{Force}	0.01	Estimated
Force activation probability for protrusive force	p_{Prot}^{Force}	0.1	Estimated
Constant for repulsion	K_{rep}	10^{-3} (kg/s^2)	Estimated
Maximum distance to apply repulsion	L_{rep}	0.05 (μm)	Estimated
Remodel rate constant	$k_{remodel}$	$0.025 s^{-1}$	Estimated
Hexagon side length	$l_{hexagon}$	$25 (\mu m)$	Estimated
Distance between membrane points	l_n	$625 (nm)$	Estimated
Minimum area for gap formation	$A_{GAP,F}$	$2 (\mu m^2)$	Estimated
Area for gap closing	$A_{GAP,C}$	$1.5 (\mu m^2)$	Estimated
Time step	Δt	$1.26 (s)$	

Table S1: Reference model parameters used in the simulation.
