# Supplementary figures and images for: Balance of mechanical forces drives endothelial gap formation and may facilitate cancer and immune-cell extravasation
Source: PLoS Comput Biol. 2019 May 2;15(5):e1006395. doi: 10.1371/journal.pcbi.1006395 (PMC6497229; doi:10.1371/journal.pcbi.1006395)

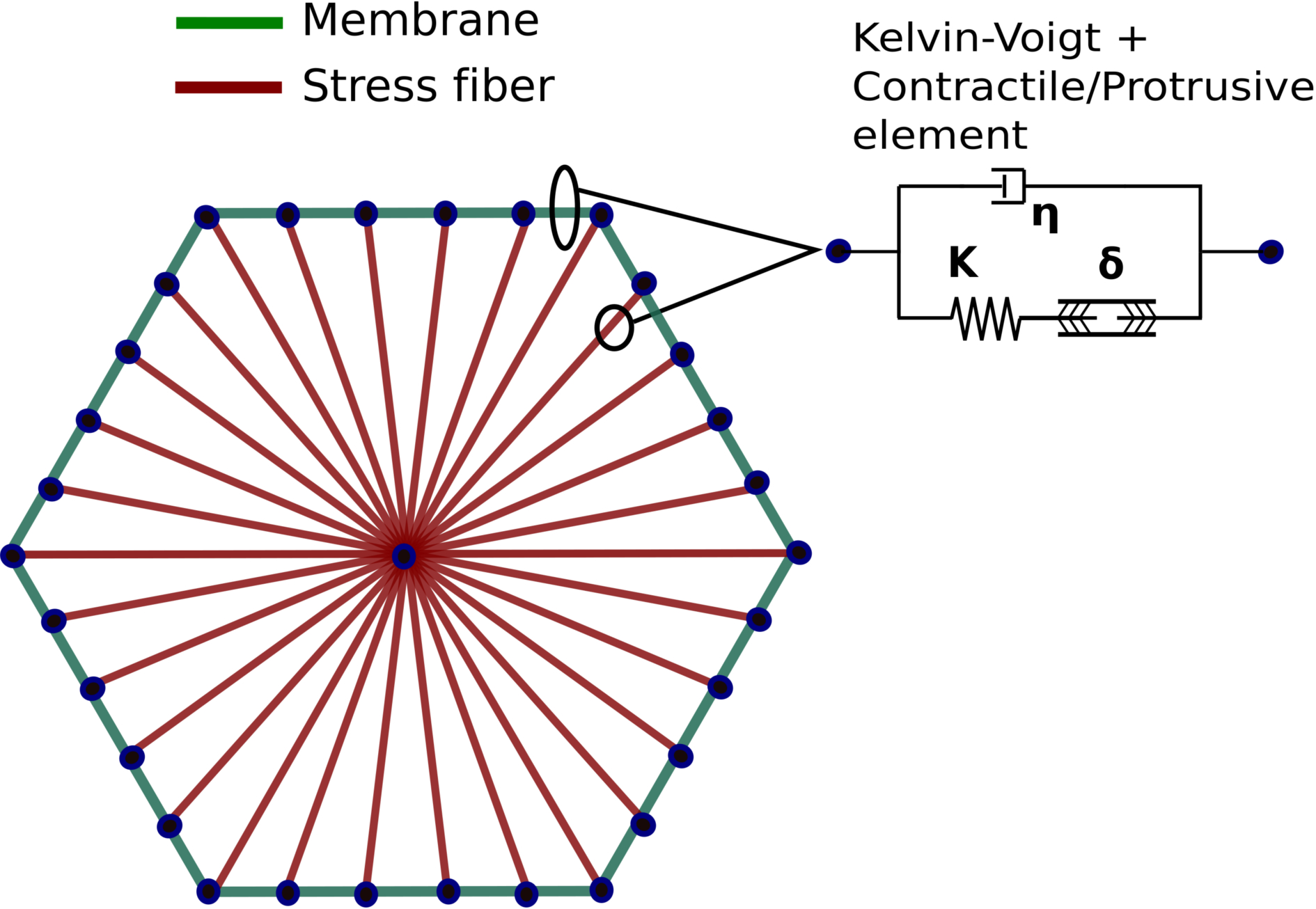

Supplement: S1 Fig — The cell is presented in an initially hexagonal form, divided into a discrete number of membrane points. Physically, our membrane elements connecting the nodes represent the combined lipid bilayer with the actin cortex. Moreover, the nodes are connected to the center by stress fiber structure. Both of them are described by Kelvin-Voigt models with a contractile/protrusive element, but both have different parameters. (TIF) [file pcbi.1006395.s003.tif]

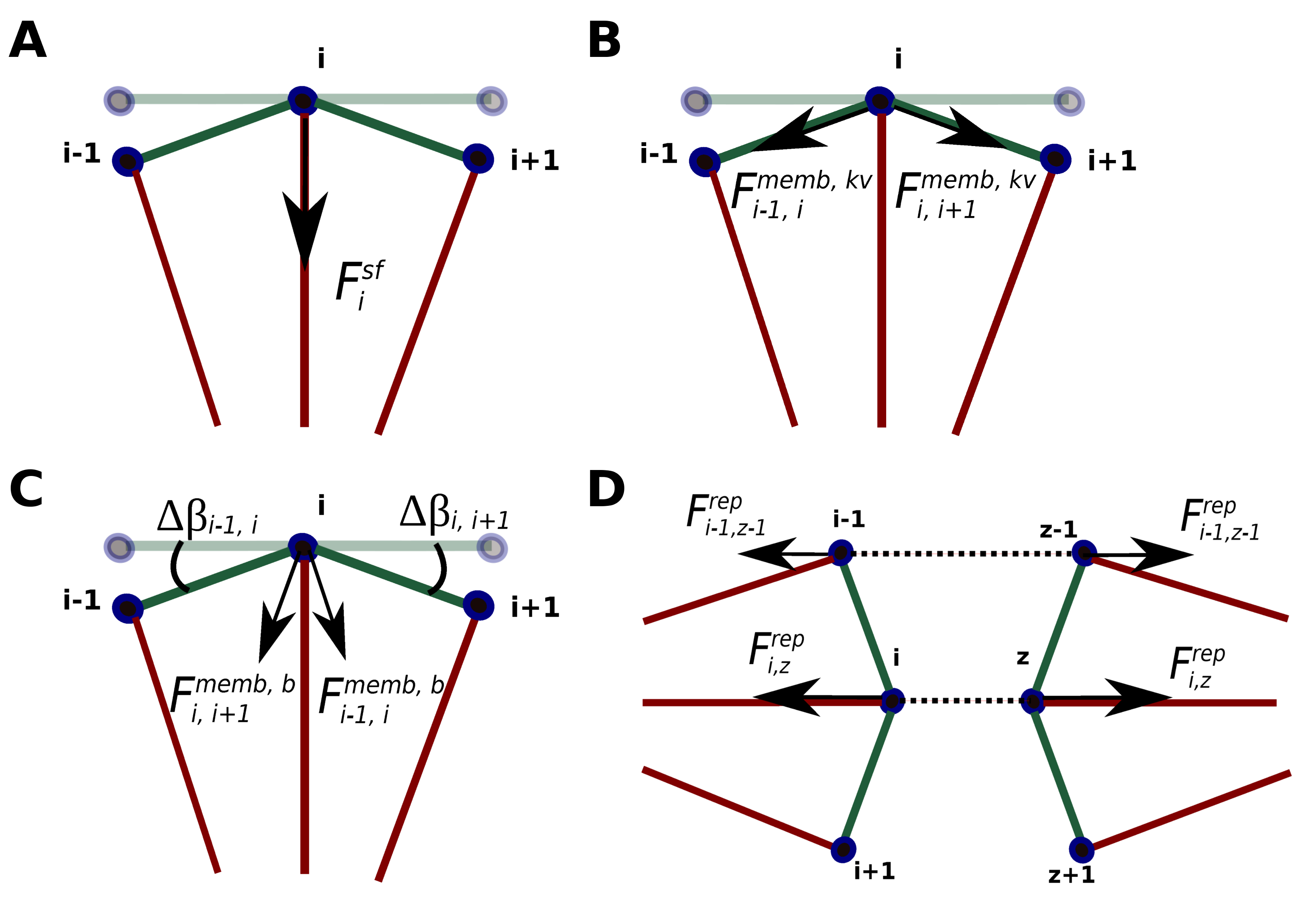

Supplement: S2 Fig — (A) Force due to stress fiber deformations. (B) Force due to membrane in-plane deformation. (C) Force due to membrane bending stiffness. (D) Force due to repulsion between membrane points of different cells. (TIF) [file pcbi.1006395.s004.tif]

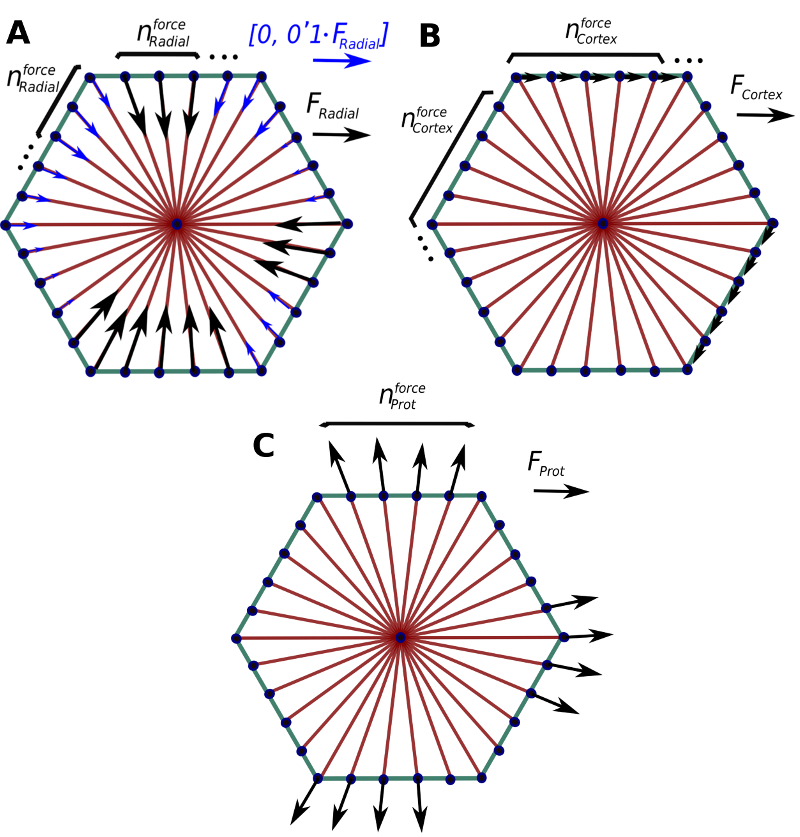

Supplement: S3 Fig — (A) and (B) Correspond to myosin forces: Radial force and Cortex force respectively. (C) Protrusive forces. (TIF) [file pcbi.1006395.s005.tif]

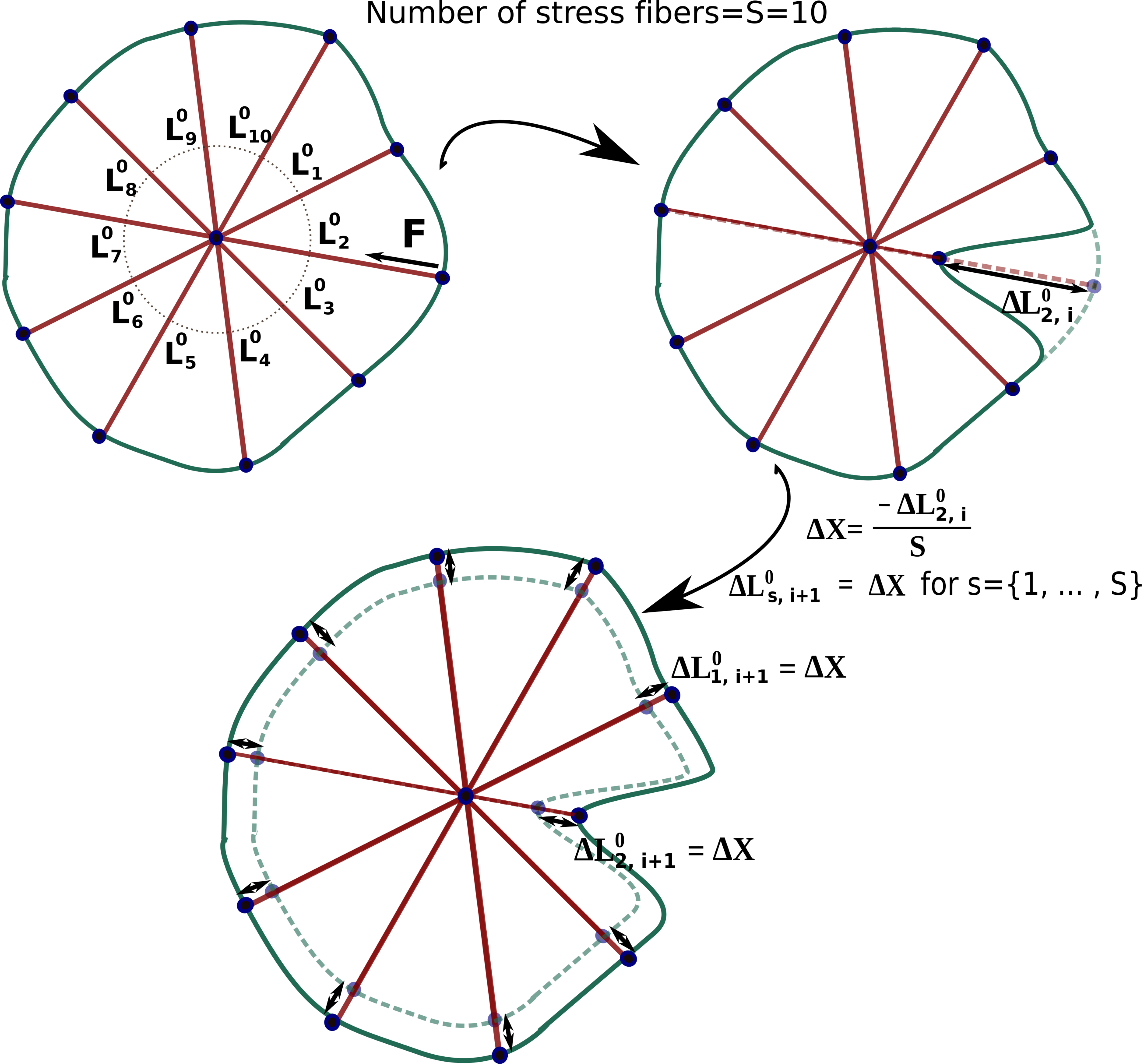

Supplement: S4 Fig — Due to myosin contractility, a change in the rest length of the stress fiber occurs accordingly to Eq. S15. This change in rest length is compensated by all the stress fibers in a proportional way. Note that only the rest lengths and not the current length of a stress fiber is modified. (TIF) [file pcbi.1006395.s006.tif]

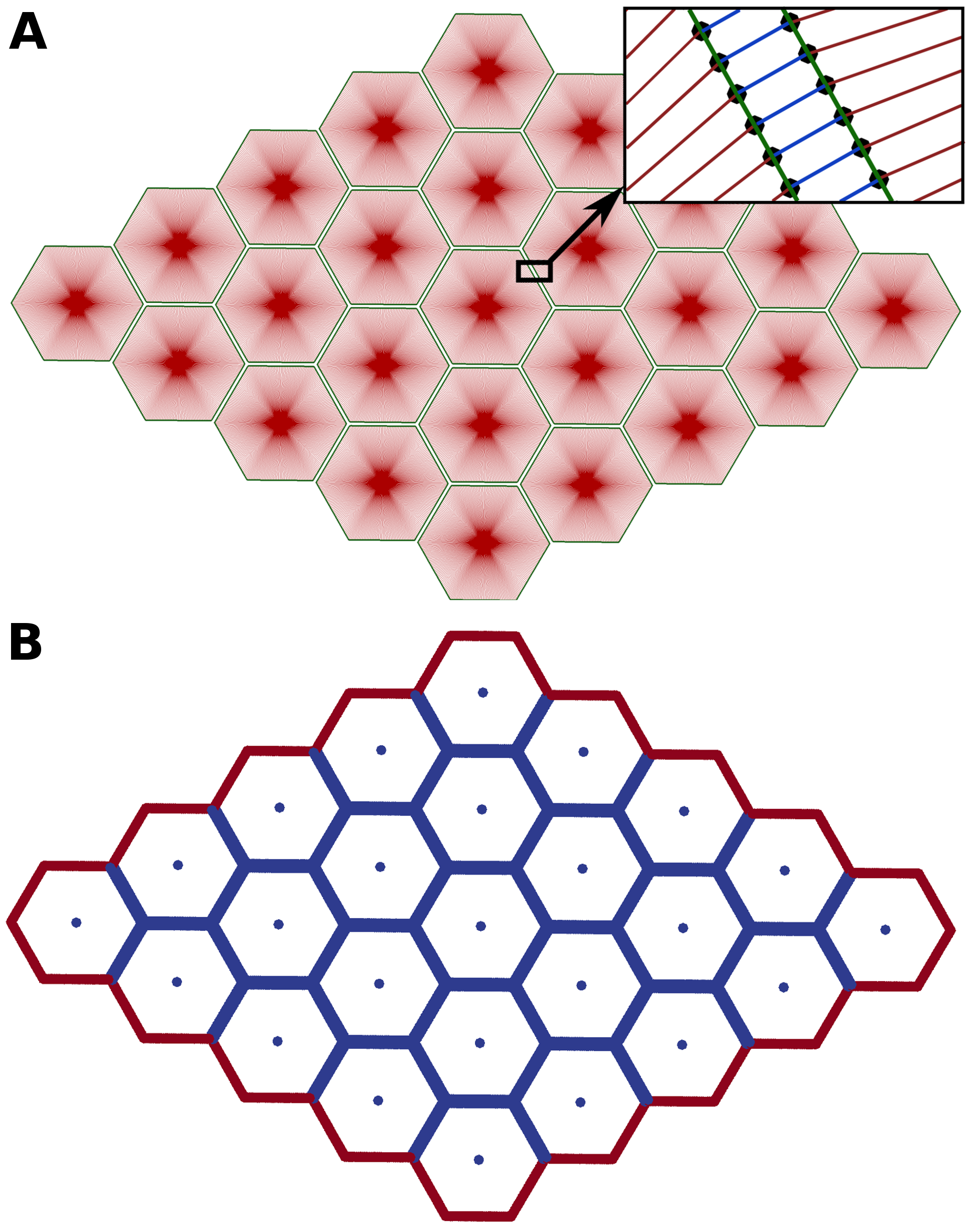

Supplement: S5 Fig — A: Cells with a hexagonal shape are in a rest state and fully bound to their neighboring cells. Cell membrane (green), stress fibers (red), cadherin complexes (blue), membrane points (black). B: Boundary conditions: Points in the boundary of the monolayer (red) are fixed. In blue are membrane points and the cell centers. (TIF) [file pcbi.1006395.s007.tif]

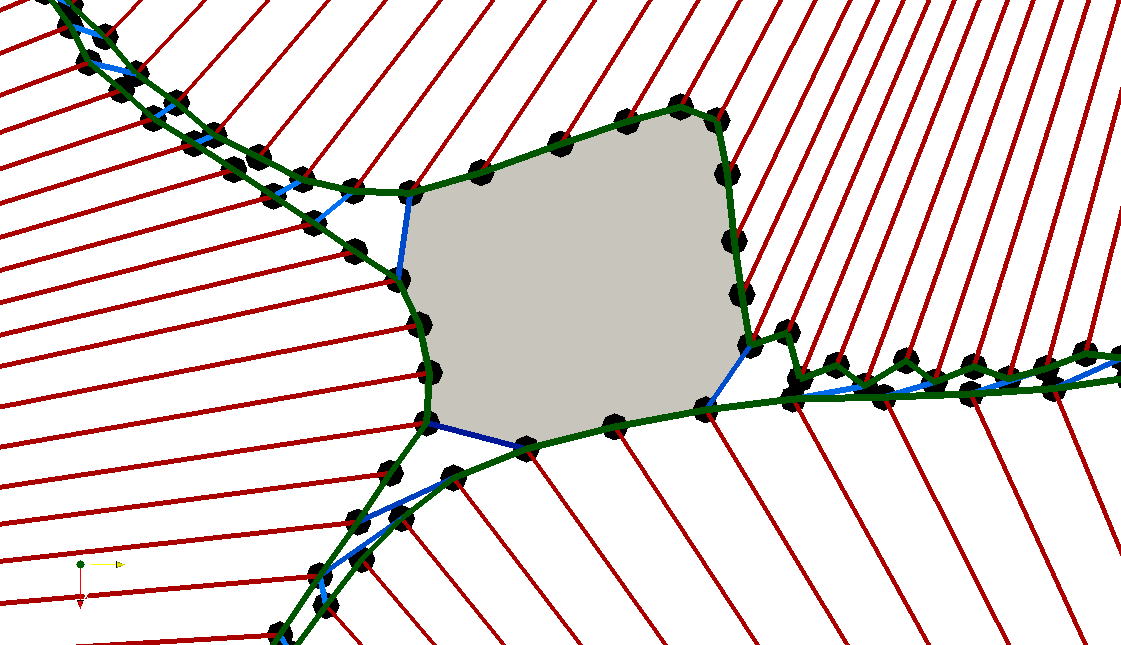

Supplement: S6 Fig — A gap (grey area) is delimited by the cell membrane (green) and the adhesion bonds binding the cells (blue). Red: cell stress fibers. Black dots: Membrane points. (TIF) [file pcbi.1006395.s008.tif]

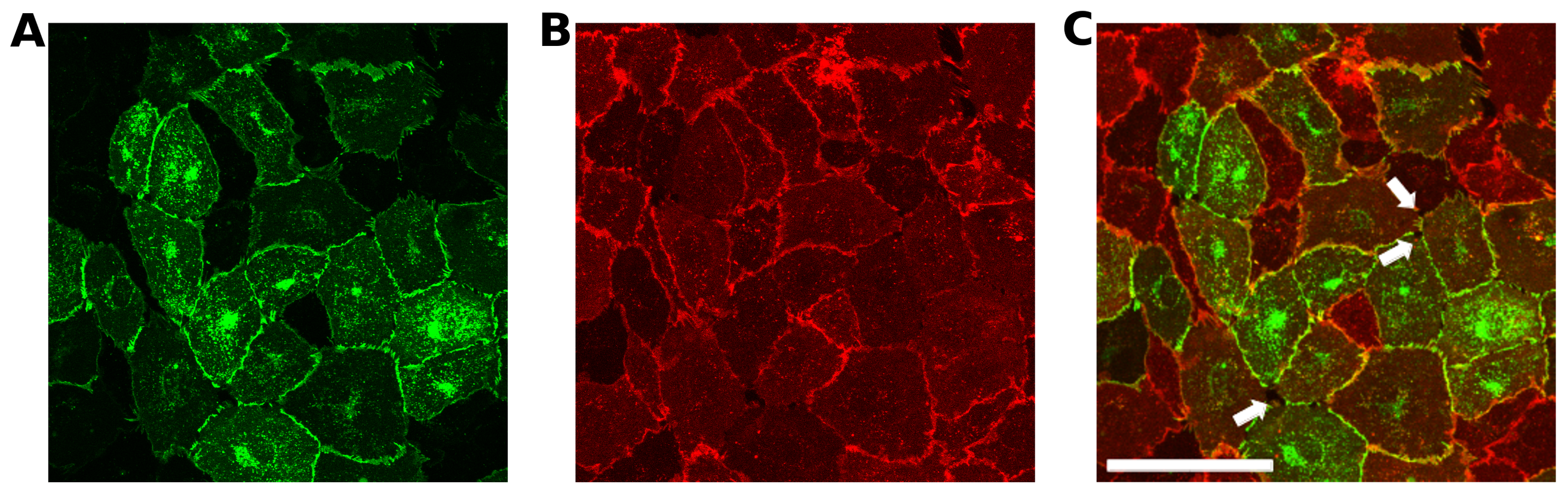

Supplement: S7 Fig — Endothelial monolayer stained with VE-cadherin (green, A) and CD31 (red, B). C: Merged image confirms that gaps observed within the VE-cadherin mediated cell-cell adhesions are also present within CD31, indicating that gaps seen in VE-cadherin are real physical gaps between the cells. Scale bar 100μm. (TIF) [file pcbi.1006395.s009.tif]

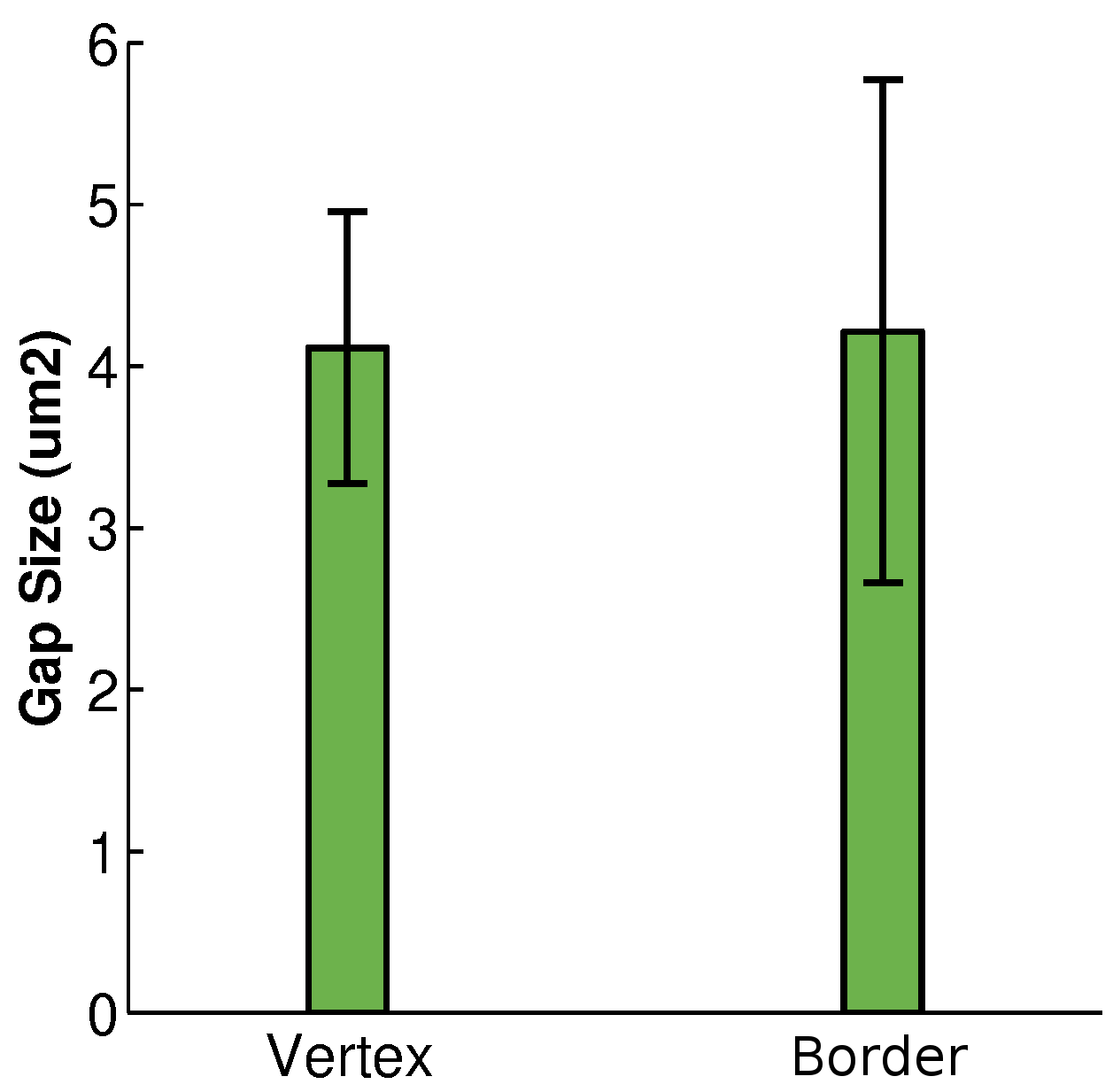

Supplement: S8 Fig — Average size of the gaps generated at the vertices and borders. Parameters are the reference values as in S1 Table and error bars correspond to standard deviation of sample = 30. (TIF) [file pcbi.1006395.s010.tif]

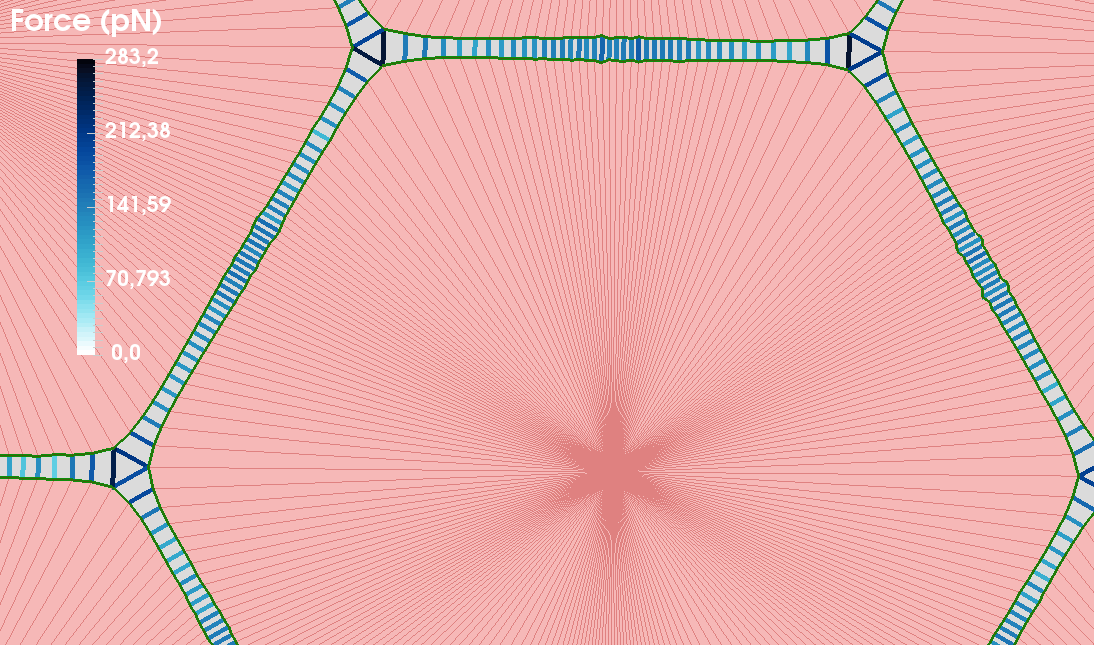

Supplement: S9 Fig — Homogeneous contractions are applied to all the hexagonal cells in the monolayer. Stresses concentrate on the adhesions at vertices, as opposed to the adhesions at border. (TIF) [file pcbi.1006395.s011.tif]

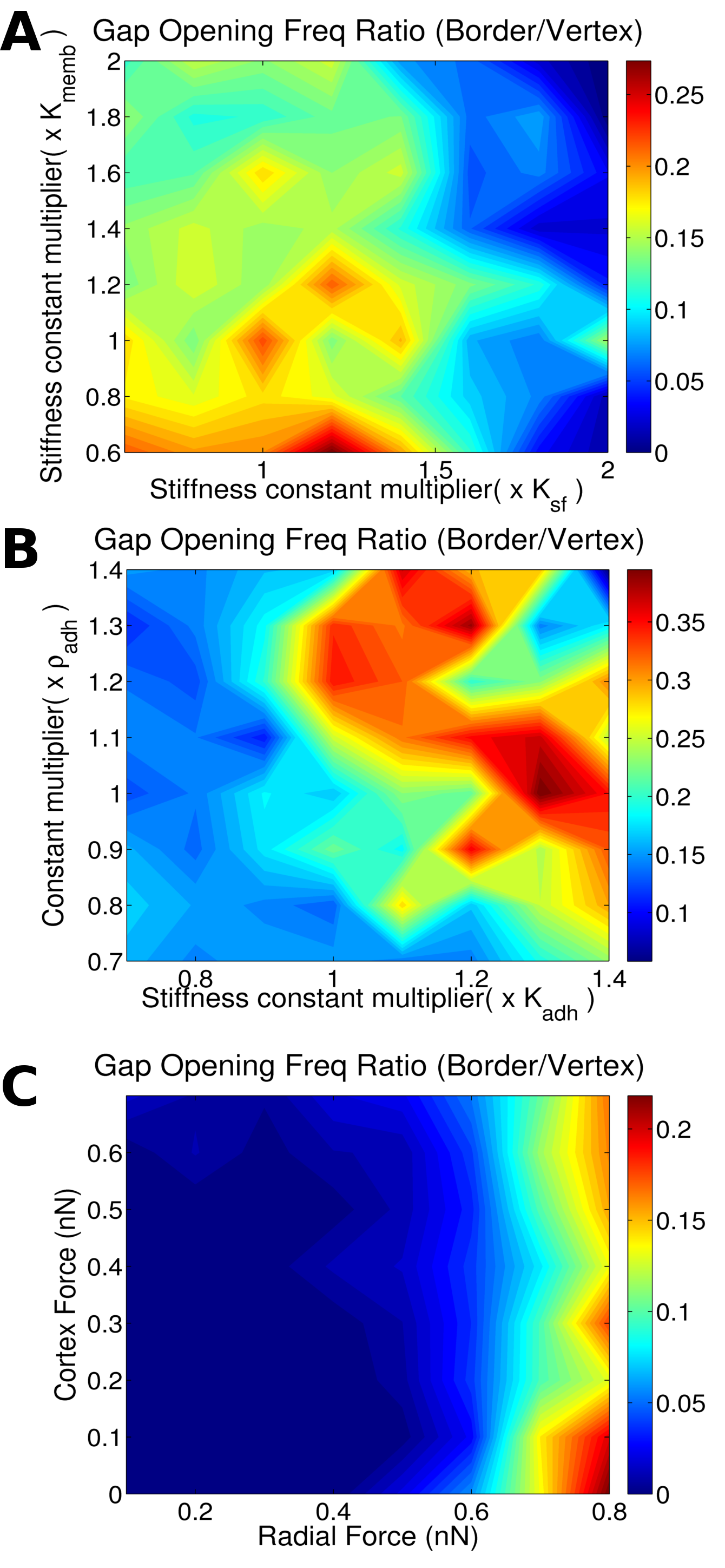

Supplement: S10 Fig — Shown is the ratio of gaps that occur at a two cell border divided by the gaps that originate at a three cell vertex. A shows results varying membrane and stress fiber stiffness. B shows properties of cell-cell junction are changed: cadherin stiffness versus cadherin density (binding rate). C shows results for varying cortical and radial force. (TIF) [file pcbi.1006395.s012.tif]

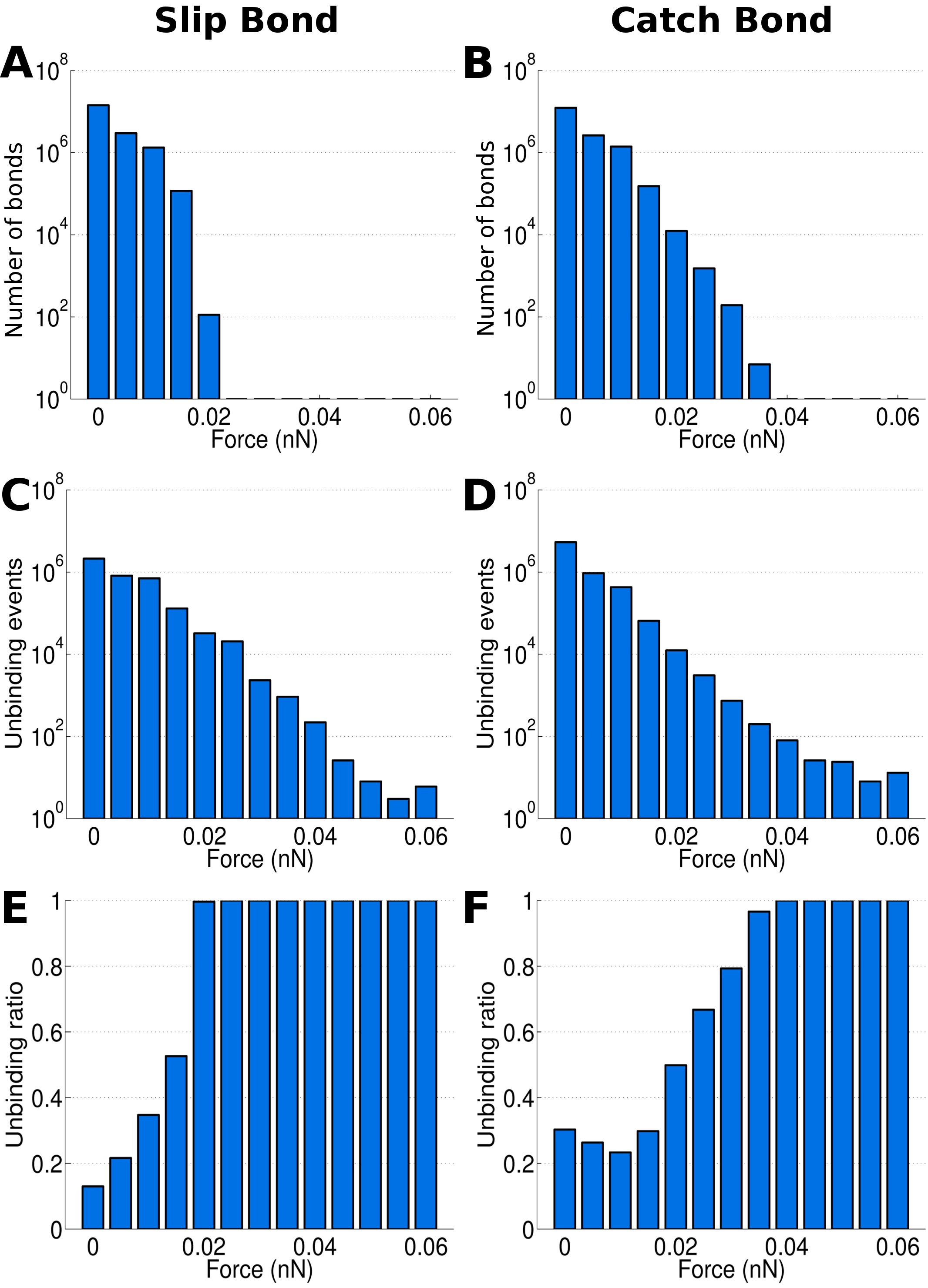

Supplement: S11 Fig — First row (A, B) shows force histogram of cadherins that are bound for slip and catch bond respectively. Second row (C, D) cadherins force at which cadherins unbind for slip and catch bond respectively. Third row (E, F) shows the ratio obtained by dividing unbound cadheins by the sum of unbound cadherins and bound cadherins (ub/(ub + b), where ub and b corresponds to unbound and bound cadherins respectively). (TIF) [file pcbi.1006395.s013.tif]

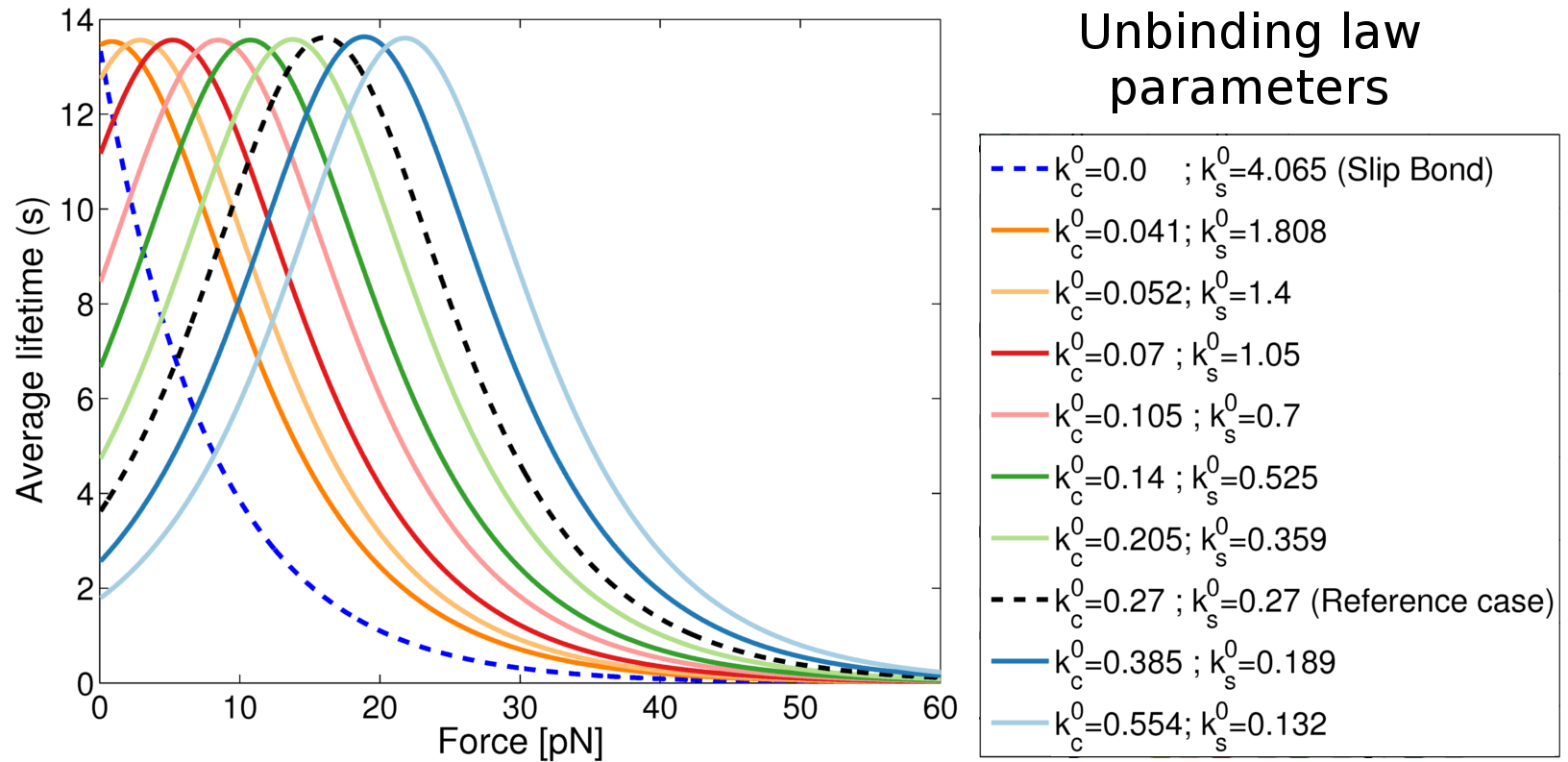

Supplement: S12 Fig — Lifetime average for the bond in dependence on the force for different unbinding laws. Legend shows the parameter variation to obtain the different curves. (TIF) [file pcbi.1006395.s014.tif]

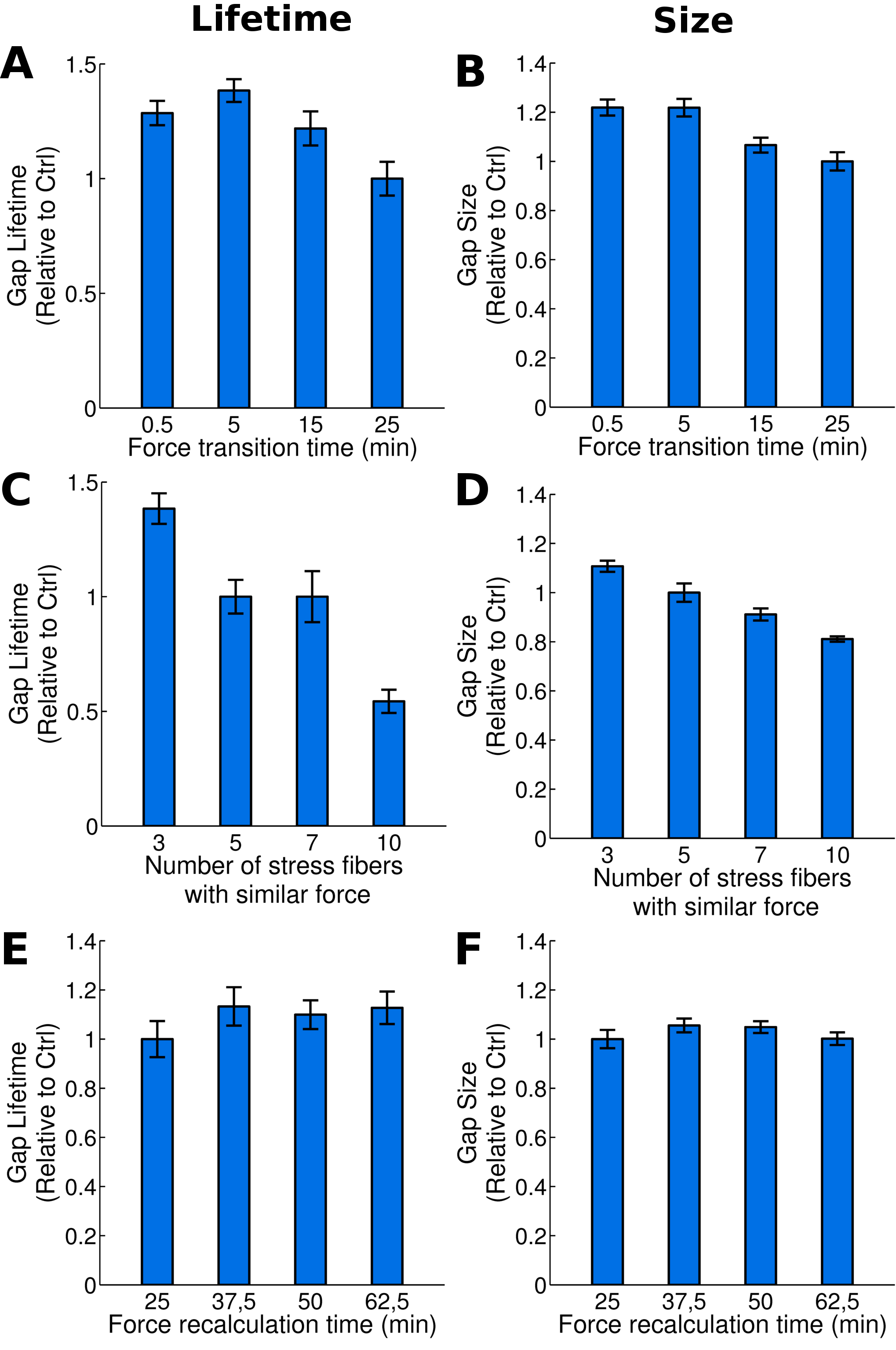

Supplement: S13 Fig — Corresponds to Fig 4. Left column corresponds to lifetime and right column to size. (A, B): Changes in the transition time of the application of the recalculated forces. Longer time means smoother force changes. (C, D) Variation in the number of stress fibers over which the same force is distributed. (E, F) Variation in force fluctuation time for all types of forces considered in the model. Error bars show to the standard error. (TIF) [file pcbi.1006395.s015.tif]

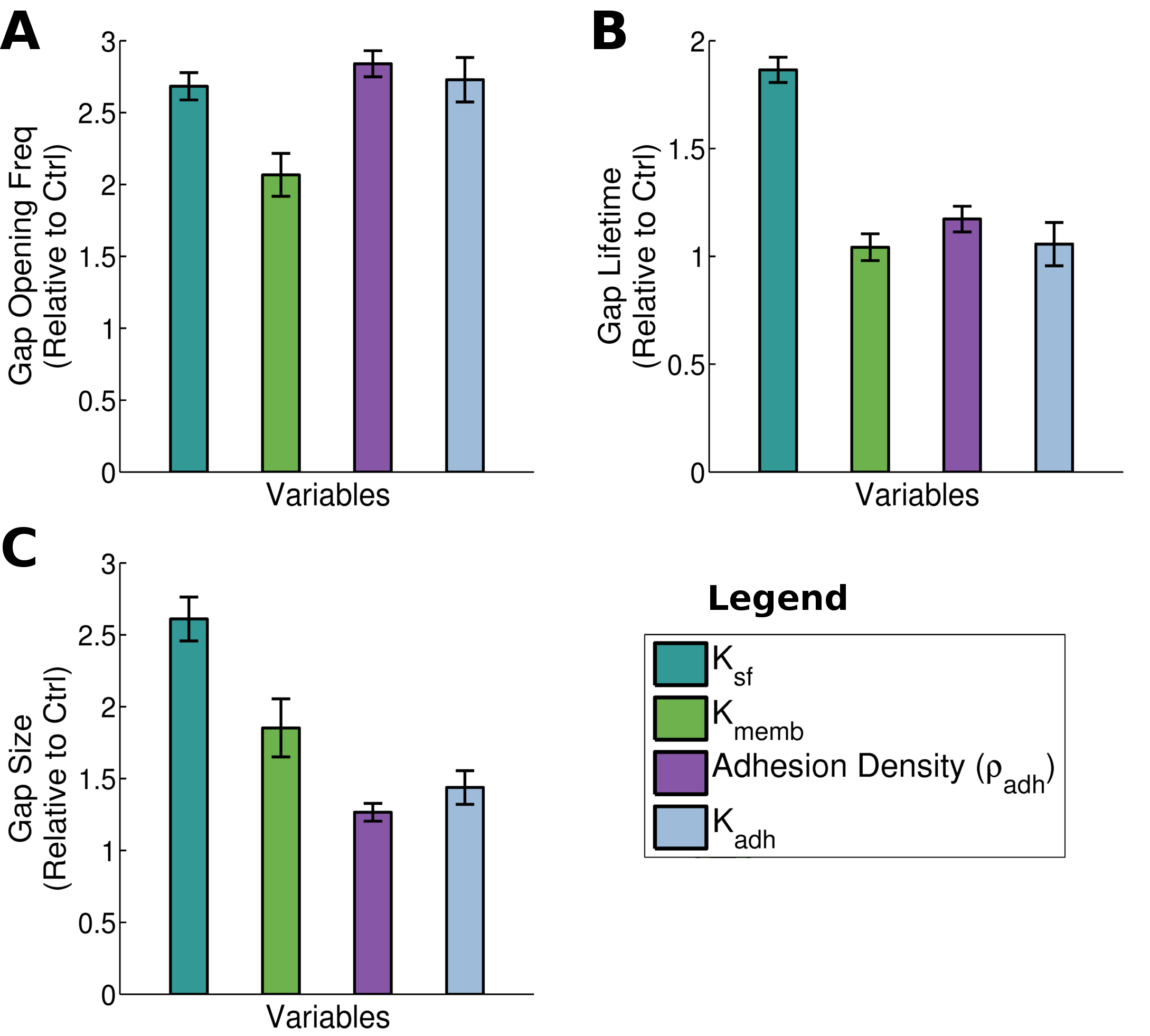

Supplement: S14 Fig — Error bars show to the standard error. All parameters have been reduced one order of magnitude (x10−1). (A) Gap opening frequency. (B) Average lifetime of the gaps. (C) Average size of the gaps. (TIF) [file pcbi.1006395.s016.tif]

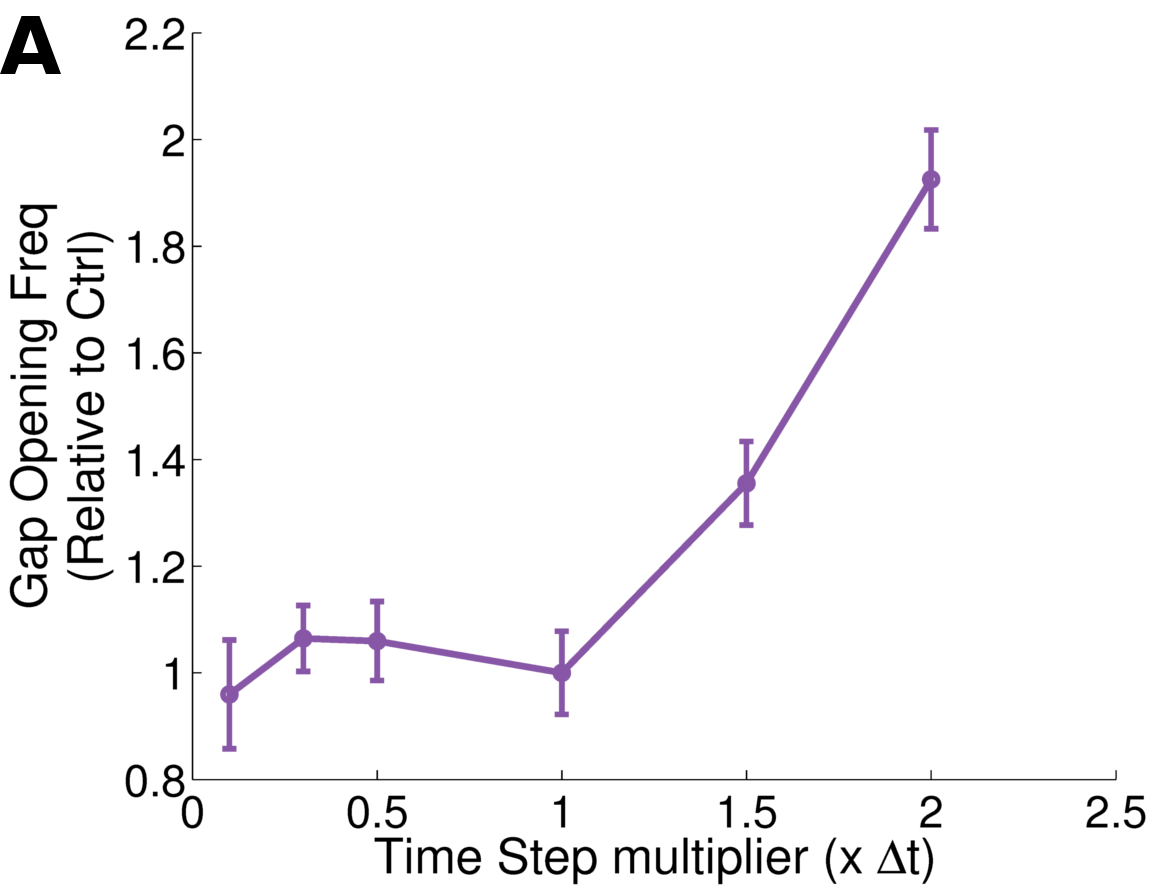

Supplement: S15 Fig — The gap opening frequency depends on the time step used in our numerical simulations. Note that the time step multiplier is relative to the reference case (multiplier = 1). Error bars are the standard error. The results confirm that the time step selected for the reference case is low enough to ensure convergence of the results. (TIF) [file pcbi.1006395.s017.tif]

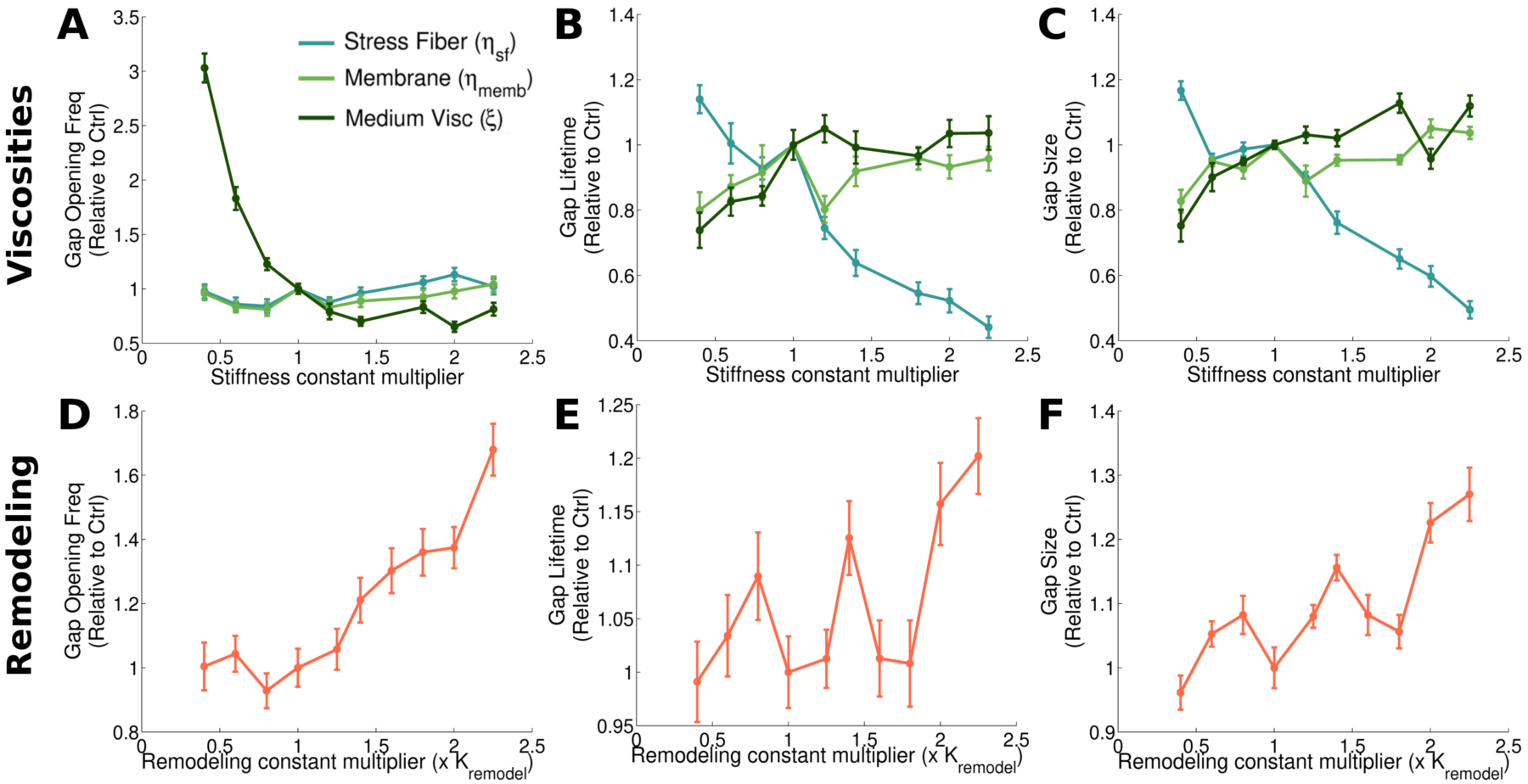

Supplement: S16 Fig — Gap opening frequency, average lifetime and size in each column. Note that the point where x and y coordinates are 1 corresponds to the reference case. Error bars represent standard error. In the first row (A, B, C), results for medium and dashpot viscosities of the stress fibers and membrane and varied. Increasing viscosity reduces node movement, stabilizing monolayer dynamics. Medium viscosity has a higher effect on gap opening dynamics since it affects the overall timescale of all mechanical parts of the model. The stress fiber dashpot strongly influences gap lifetime and size; this is similar to the dominating effect of stress fiber stiffness over membrane stiffness on gap lifetime and size (Fig 3A and 3B). The second row (D, E, F) shows the effect of varying the constant for remodeling rate. Increasing the remodeling rate implies that cells are able to adapt their permanent shapes faster in response to deformations. Therefore, the frequency of gap openings increases with the remodeling rate (D). The gap lifetime and size broadly also increase, but less strongly then the opening frequency. (TIF) [file pcbi.1006395.s018.tif]
